# Alloreactive T Cell Receptor Diversity against Structurally Similar or Dissimilar HLA-DP Antigens Assessed by Deep Sequencing

**DOI:** 10.3389/fimmu.2018.00280

**Published:** 2018-02-19

**Authors:** Esteban Arrieta-Bolaños, Pietro Crivello, Maximilian Metzing, Thuja Meurer, Müberra Ahci, Julie Rytlewski, Marissa Vignali, Erik Yusko, Peter van Balen, Peter A. Horn, J. H. Frederik Falkenburg, Katharina Fleischhauer

**Affiliations:** ^1^Institute for Experimental Cellular Therapy, University Hospital Essen, Essen, Germany; ^2^Adaptive Biotechnologies, Seattle, WA, United States; ^3^Department of Hematology, Leiden University Medical Center, Leiden, Netherlands; ^4^Institute for Transfusion Medicine, University Hospital Essen, Essen, Germany; ^5^German Cancer Consortium (DKTK), Heidelberg, Germany

**Keywords:** T-cell alloreactivity, T-cell receptor repertoire, next-generation sequencing, human leukocyte antigen-DPB1, permissive mismatches

## Abstract

T cell alloreactivity is mediated by a self-human leukocyte antigen (HLA)-restricted T cell receptor (TCR) repertoire able to recognize both structurally similar and dissimilar allogeneic HLA molecules (i.e., differing by a single or several amino acids in their peptide-binding groove). We hypothesized that thymic selection on self-HLA molecules could have an indirect impact on the size and diversity of the alloreactive response. To test this possibility, we used TCR Vβ immunophenotyping and immunosequencing technology in a model of alloreactivity between self-HLA selected T cells and allogeneic HLA-DPB1 (DPB1) differing from self-DPB1*04:02 by a single (DPB1*02:01) or several (DPB1*09:01) amino acids in the peptide-binding groove. CD4+ T cells from three different self-DPB1*04:01,*04:02 individuals were stimulated with HeLa cells stably transduced with the relevant peptide processing machinery, co-stimulatory molecules, and HLA-DP. Flow cytometric quantification of the DPB1-specific T cell response measured as upregulation of the activation marker CD137 revealed significantly lower levels of alloreactivity against DPB1*02:01 compared with DPB1*09:01 (mean CD4+CD137+ frequency 35.2 ± 9.9 vs. 61.5 ± 7.7%, respectively, *p* < 0.0001). These quantitative differences were, however, not reflected by differences in the breadth of the alloreactive response at the Vβ level, with both alloantigens eliciting specific responses from all TCR-Vβ specificities tested by flow cytometry, albeit with higher levels of reactivity from most Vβ specificities against DPB1*09:01. In line with these observations, *TCRB*-CDR3 immunosequencing showed no significant differences in mean clonality of sorted CD137+CD4+ cells alloreactive against DPB1*02:01 or DPB1*09:01 [0.39 (0.36–0.45) and 0.39 (0.30–0.46), respectively], or in the cumulative frequencies of the 10 most frequent responding clones (55–67 and 58–62%, respectively). Most of the clones alloreactive against DPB1*02:01 (68.3%) or DPB1*09:01 (75.3%) were characterized by low-abundance (i.e., they were not appreciable among the pre-culture T cells). Interestingly, however, their cumulative frequency was lower against DPB1*02:01 compared with DPB1*09:01 (mean cumulative frequency 35.3 vs. 50.6%, respectively). Our data show that, despite lower levels of alloreactivity, a similar clonal diversity can be elicited by structurally similar compared with structurally dissimilar HLA-DPB1 alloantigens and demonstrate the power of *TCRB* immunosequencing in unraveling subtle qualitative changes not appreciable by conventional methods.

## Introduction

T cell alloreactivity against the human leukocyte antigen (HLA) system is a major barrier to successful transplantation of organs and stem cells. Alloreactivity is mediated by a T cell repertoire shaped by thymic selection to be self-HLA restricted, but at the same time capable of recognizing non-self-HLA. The fact that a large proportion of T cells is capable of recognizing previously unseen alloantigens (1, 2) remains an enigma of T cell biology. T cell alloreactivity involves a complex interplay between the polymorphic foreign HLA molecule, the peptides being presented by it, and the self T cell receptor (TCR) (3). Although the relative contribution of each of these components to alloreactivity is not completely understood, it is conceivable that amino acid changes in the peptide-binding region of a given HLA molecule may ensue changes in the biochemical and/or structural properties of the binding pockets, thereby impacting the amino acid sequence and/or conformation of peptides able to be presented by it. Alternatively, these amino acid changes could directly affect the interaction between the HLA molecule and the TCR (4). Whether the number of amino acid differences in the peptide-binding region of the HLA molecule results in higher or lower levels of alloreactivity and whether this arises from a broader or narrower alloreactive T cell response is a matter of debate.

Of note, it has been shown that even a single amino acid difference in the peptide-binding region of HLA class I molecules HLA-B*44:02 and B*44:03 can elicit a T cell response sufficient to cause allograft rejection (5) or graft-vs.-host disease (GvHD) (6) after hematopoietic stem cell transplantation (HSCT). In addition, *in vitro* measurements of the patient–donor immune response before HSCT, mainly based on direct recognition of mismatched HLA class I antigens, have suggested that the number of amino acid differences is inversely correlated with the amount of direct T cell allorecognition (7), although this concept was not supported by clinical associations with HSCT outcome (8). Our understanding, control, and capacity to harness alloreactivity in the transplantation setting are still incomplete.

HLA-DPB1 (DPB1) represents an attractive model for the study of alloreactive responses to HLA molecules. Previous work by us (9) and others (10, 11) has shown differential alloreactivity to allogeneic DPB1 according to a functional classification of its different allelic variants (12). Amino acid changes resulting in structural and functional dissimilarities between DPB1 alleles were shown to have a strong median impact on alloreactive responses to these molecules (13), allowing for the classification of DPB1 mismatches as permissive (structural similarity and low alloreactivity) or non-permissive (structural dissimilarity and higher alloreactivity) in the clinical setting (14, 15). Importantly, the classification of a mismatch as permissive or non-permissive depends on the self-HLA background of the responder, following the concepts of thymic T cell education (16). However, direct evidence for the hypothesis that thymic selection on self-alleles has an indirect impact on the size and diversity of the alloreactive response has yet to be obtained. Here, we have sought to fill this gap by characterizing the alloreactive TCR diversity from self-DPB1*04:01,*04:02 individuals against alloantigens carrying a single (DPB1*02:01) or multiple (DPB1*09:01) amino acid differences in the peptide-binding groove using a unique system of single-DPB1 allele-expressing cells, and TCR Vβ immunophenotype and deep immunosequencing of the *TCRB* gene.

## Materials and Methods

### Subjects and Cells

Buffy coats from three healthy blood donors were obtained in order to isolate peripheral blood mononuclear cells (PBMC) by Ficoll centrifugation. All blood donors had been typed as self-DPB1*04:01,*04:02 by standard molecular methods and were CMV seronegative. Demographic details of each subject are presented in Table 1. PBMC were then used to isolate untouched CD4+ T cells *via* magnetic beads according to the manufacturer’s instructions (Miltenyi Biotec GmbH, Bergisch Gladbach, Germany). Purified CD4+ T cells (average 97.7%, range: 96.7–98.3% of live cells, with a CD8+ mean content of 0.02%, range: 0.01–0.04%) were used as responders in coculture with stimulating cells as described subsequently. All participants gave informed consent, and this study was approved by the local ethics committee of University Hospital Essen.

**Table 1:** HLA, CMV, and demographic data for the healthy subjects used in this study.

Subject	DPB1 typing	CMV serostatus	Age (years)	Gender
R1	*04:01,*04:02	Negative	67	Male
R2	*04:01,*04:02	Negative	56	Male
R3	*04:01,*04:02	Negative	21	Female

*CMV, cytomegalovirus; HLA, human leukocyte antigen; R, responder*.

### Expansion of DPB1-Specific CD4+ Cells

In order to expand DPB1-specific alloreactive T cells, purified CD4+ cells from each donor were cocultured with HeLa cells expressing single specific HLA-DP molecules as described previously (17, 18). In brief, HeLa cells, which normally do not express HLA class II molecules, were retrovirally transduced with specific HLA-DP molecules (DPB1 and the naturally associated DPA1 genes) and the necessary machinery for HLA class II antigen presentation (HLA-DM and invariant chain) and co-stimulation (CD80) and used as antigen-presenting cells. Expression of the DP heterodimer was confirmed by flow cytometry (anti-DP clone B7/21; Leinco Technologies, Inc., St. Louis, MO, USA) with comparable levels among different transduced cells (data not shown). Purified CD4+ T cells (typically 1.2 million) were cultured in RPMI (c.c.pro GmbH, Oberdorla) supplemented with l-glutamine (2 mM), penicillin–streptomycin (100 ng/mL), and 10% human AB serum at a ratio of 3:1 with irradiated (100 Gy) HeLa cells in the presence of 50 U/mL IL-2 in 24-well plates. Parallel cocultures were set up for each subject using HeLa expressing DPB1*02:01 (one peptide-binding groove amino acid difference with DPB1*04:02) or DPB1*09:01 (10 peptide-binding groove amino acid differences with DPB1*04:02). After 15 days, expanded CD4+ T cells were rechallenged for 24 h with HeLa expressing the allogeneic stimulator DPB1 at the same ratio in order to assess the alloreactive response in terms of CD137+ (i.e., activated) T cells by flow cytometry (19). HeLa cells transduced to express one of the donors’ self-HLA DPB1 molecules (i.e., *04:01) were used to determine background activation levels (average 4.1%). After rechallenge, T cells were stained with fluorescently labeled antibodies against CD3 (clone UCHT1, Beckman Coulter, Marseille, France), CD4 (clone SK3, BD Biosciences, Heidelberg, Germany), CD8 (clone B9.11, Beckman Coulter, Marseille, France), and CD137 (clone 4B4-1, BD Biosciences, Heidelberg, Germany), and the proportion of CD137+CD4+ cells was measured on a Gallios flow cytometer (Beckman Coulter GmbH, Krefeld, Germany). CD137+CD4+ cells were then assessed for their TCR diversity as explained subsequently.

### TCR Vβ Immunophenotype

T cell receptor diversity of anti-DPB1 T cell cultures was assessed in total and CD137+CD4+ cells at the Vβ level by flow cytometry using the IOTest^®^ Beta Mark TCR Vβ Repertoire kit (Beckman Coulter, Marseille, France). For this, one million cultured CD4+ cells were restimulated with the specific HeLa transduced cells for 24 h. Then, the T cells were harvested and stained with subset markers as indicated earlier and the kit’s eight Vβ antibody cocktails according to manufacturer’s instructions. The frequency of each of the targeted Vβ specificities was recorded in the reactive (CD4+CD137+) fraction. In addition, the proportion of CD137+ cells (responsiveness) among all CD4+ cells expressing each of the Vβ specificities was quantified. Pre-culture-isolated CD4+ cells from each subject were also analyzed in parallel as baseline control.

### TCR Immunosequencing

Next-generation sequencing-based high-throughput TCR analysis (TCR immunosequencing) was carried out in DPB1 alloreactive T cells. For this, two million cultured CD4+ cells were restimulated with the specific HeLa transduced cells for 24 h, after which the T cells were harvested and sorted for CD137 positivity (average purity 93.2%) using magnetic bead technology according to manufacturer’s instructions (Miltenyi Biotec GmbH, Bergisch Gladbach). Enrichment for DPB1-specific alloreactive T cells was confirmed by interferon-γ Elispot assays (data not shown). Genomic DNA from sorted CD137+CD4+ cells and pre-culture-isolated CD4+ samples from each subject as baseline controls was subsequently extracted using a DNeasy Blood & Tissue Kit (QIAGEN GmbH, Hilden, Germany). DNA from each cultured and pre-culture sample was sequenced to determine *TCRB* complementarity-determining region 3 (CDR3) rearrangements using the immunoSEQ*^®^* Assay from Adaptive Biotechnologies (Seattle, WA, USA) as described previously (20, 21). Briefly, a multiplex PCR system based on forward primers targeting 54 *TRBV* segments and reverse primers targeting 13 *TRBJ* segments was used to amplify the CDR3 region of the *TCRB* locus. The PCR products were sequenced on an Illumina HiSeq System, and reads of 87 base pairs covering the CDR3 region were obtained. Sequence data were preprocessed to remove PCR and sequencing errors in the primary sequence. CDR3 regions were defined based on alignments to sequences in the international ImMunoGeneTics information system^®^ (22). All cultured samples were analyzed at survey resolution (targeting 60,000 T cell genomes), while pre-culture samples were analyzed at deep resolution (targeting 200,000 T cell genomes). Average input DNA was 218.4 ng (range 137.9–400) for CD137+CD4+ cells and 1,200 ng for pre-culture CD4+ cells, respectively. The number of templates (total T cells) and the number of rearrangements (unique T cells) in each sample were estimated based on synthetic template pools as previously described (21).

### Diversity Metrics and Statistical Analyses

Immunosequencing data generated for each sample were analyzed for their TCR diversity in terms of clonality and richness. Clonality was calculated as 1-Pielou’s evenness (23), which is a measure of how uniformly distributed the repertoire is, and it is computed as normalized Shannon’s Entropy. Clonality values approaching 0 indicate that every rearrangement is present at nearly identical frequency (i.e., less variation in abundance), whereas values approaching 1 indicate a very skewed distribution of frequencies (i.e., more variation in abundance). Richness, a measure of the number of different species in a repertoire was assessed using the Daley–Smith estimate (24), a non-parametric empirical Bayes estimator of repertoire richness based on extrapolation of the rarefaction curve to 10 times the actual sample size. TCR clone sharing between samples was assessed by overlap and differential abundance analyses (25), and repertoire similarity was assessed by Morisita’s index, a population overlap metric relating the dispersion of clones in the samples (26). The abundance of individual clones was defined by assessing their presence and frequency in pre-culture and cultured samples, and low abundance clones were defined as those seen in cultured samples but undetected in the pre-culture repertoire. CDR3 immunosequencing data were analyzed using custom bioinformatics tools [R version 3.3.2 (27) and RStudio version 1.0.136 (28)] and the immunoSEQ Analyzer^®^ (Adaptive Biotechnologies, Seattle, WA, USA). Alloreactivity levels against DPB1*02:01 and DPB1*09:01 and pre-culture sample groups were compared using *t*-tests, and *p*-values < 0.01 were considered statistically significant. Statistical analyses were performed using Prism (version 6.05, GraphPad Software Inc., La Jolla, CA, USA).

## Results

### Alloreactive Response against Similar or Dissimilar DPB1 Alleles

**Figure 1:**
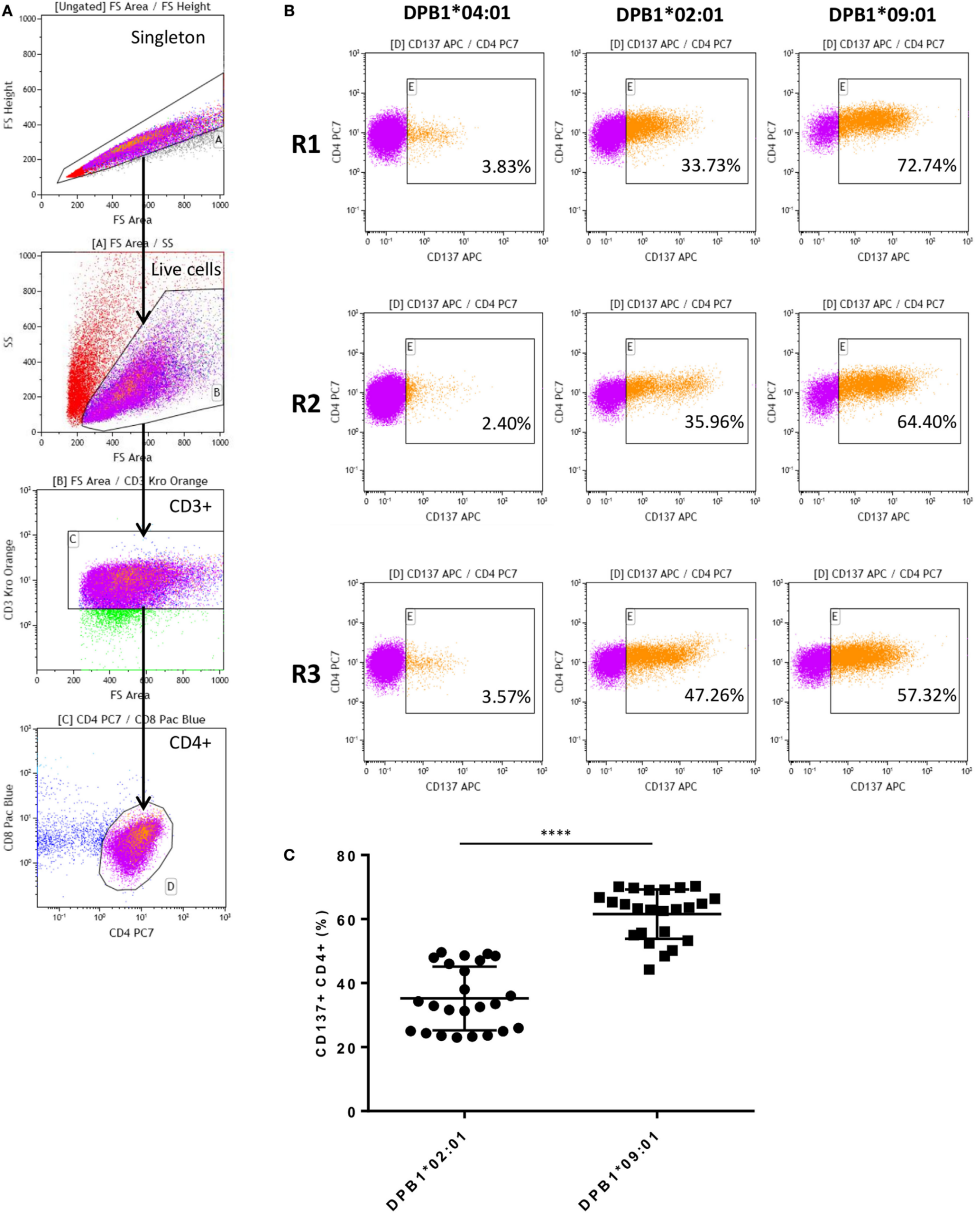
Alloresponses from self-DPB1*04:01,*04:02 individuals against HLA-DPB1*02:01 and DPB1*09:01 result in stronger responses against the latter. Isolated untouched CD4+ T cells from the three self-DPB1*04:01/*04:02 responders (R) were cocultured with HeLa cells expressing DPB1*02:01 or DPB1*09:01. After 2 weeks, expanded alloreactive T cells were restimulated for 24 h against HeLa cells expressing the original DPB1 alloantigen or an autologous allele, and the degree of T cell response was quantified as the proportion of CD4+CD137+ cells. **(A)** Gating strategy: cells are gated on the live cells and CD3+CD4+ cells. **(B)** Representative responses from each of the subjects. Left panels, background response of alloreactive cells against the autologous allele DPB1*04:01; middle panels, response against allogeneic DPB1*02:01; and right panels, response against allogeneic DPB1*09:01. **(C)** The specific response against each allele was quantified in parallel repeats (*n* = 8) for each subject after deduction of the background levels of CD137 positivity against HeLa expressing the autologous allele (average 4.11%). Mean response (and SD) against DPB1*02:01 and DPB1*09:01 was 35.2% (9.95) and 61.54% (7.69), respectively (*p* < 0.0001).

After coculture with DPB1-transduced HeLa cells, alloreactive T cells were obtained for all three responders studied against the stimulator alloantigen with either a single (DPB1*02:01, DPA1*01:03) or 10 (DPB1*09:01, DPA1*02:01) amino acid differences compared to self-DPB1*04:02. Levels of alloreactivity in our cultured samples were measured based on CD137 upregulation upon restimulation with the relevant alloantigen, and deduction of background levels of the marker against one of the self-alleles (i.e., DPB1*04:01, DPA1*01:03). Figure 1 shows the gating strategy, representative CD137 upregulation plots for each responder, and overall results. The response against DPB1*02:01 was significantly lower than that against DPB1*09:01, with mean percentages of alloreactive CD137+CD4+ of 35.2% (range 23.0–49.6%) and 61.54% (range 44.2–70.2%), respectively.

### Diversity of T Cell Responses against DPB1 Alleles at the TCR-Vβ Level

**Figure 2:**
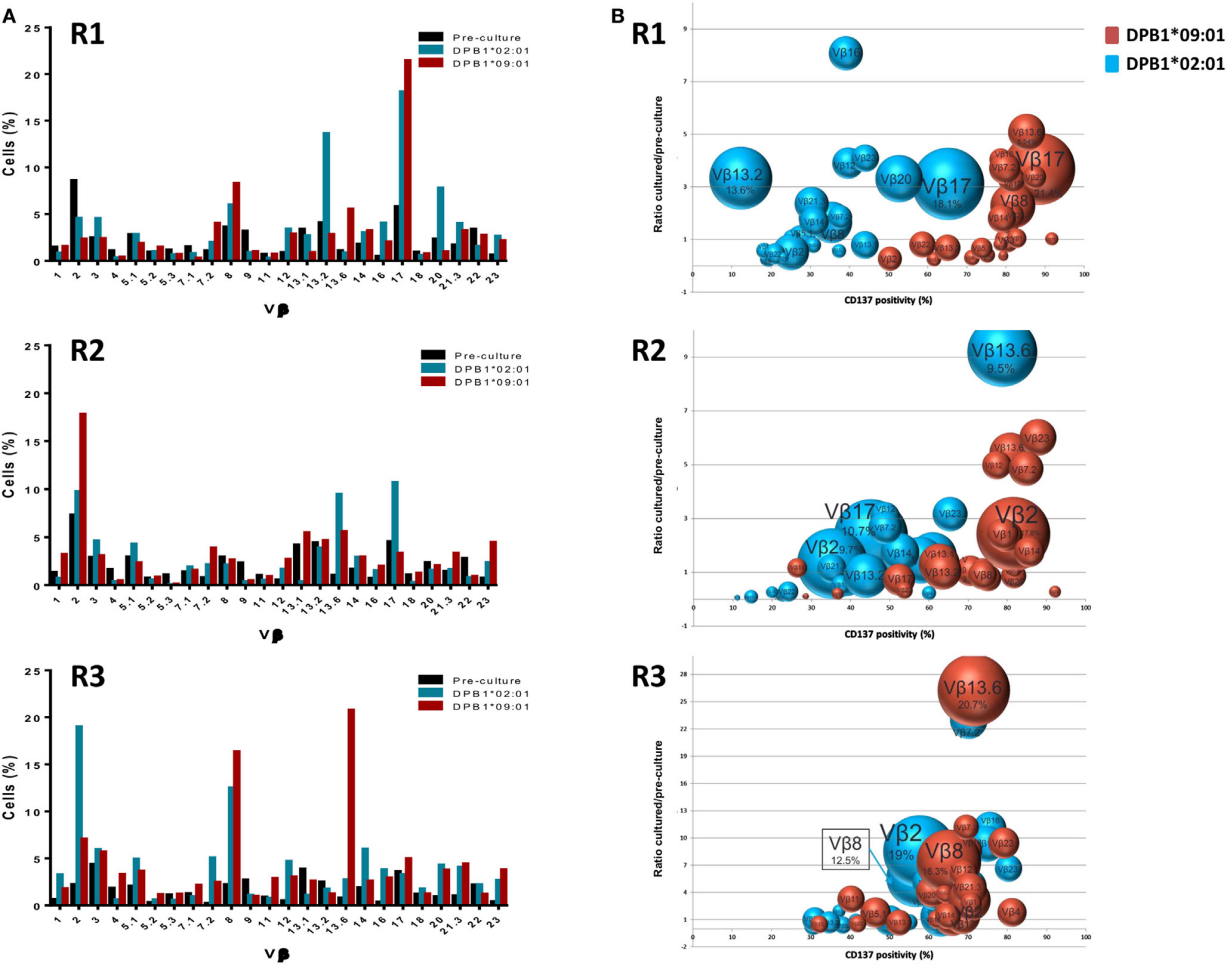

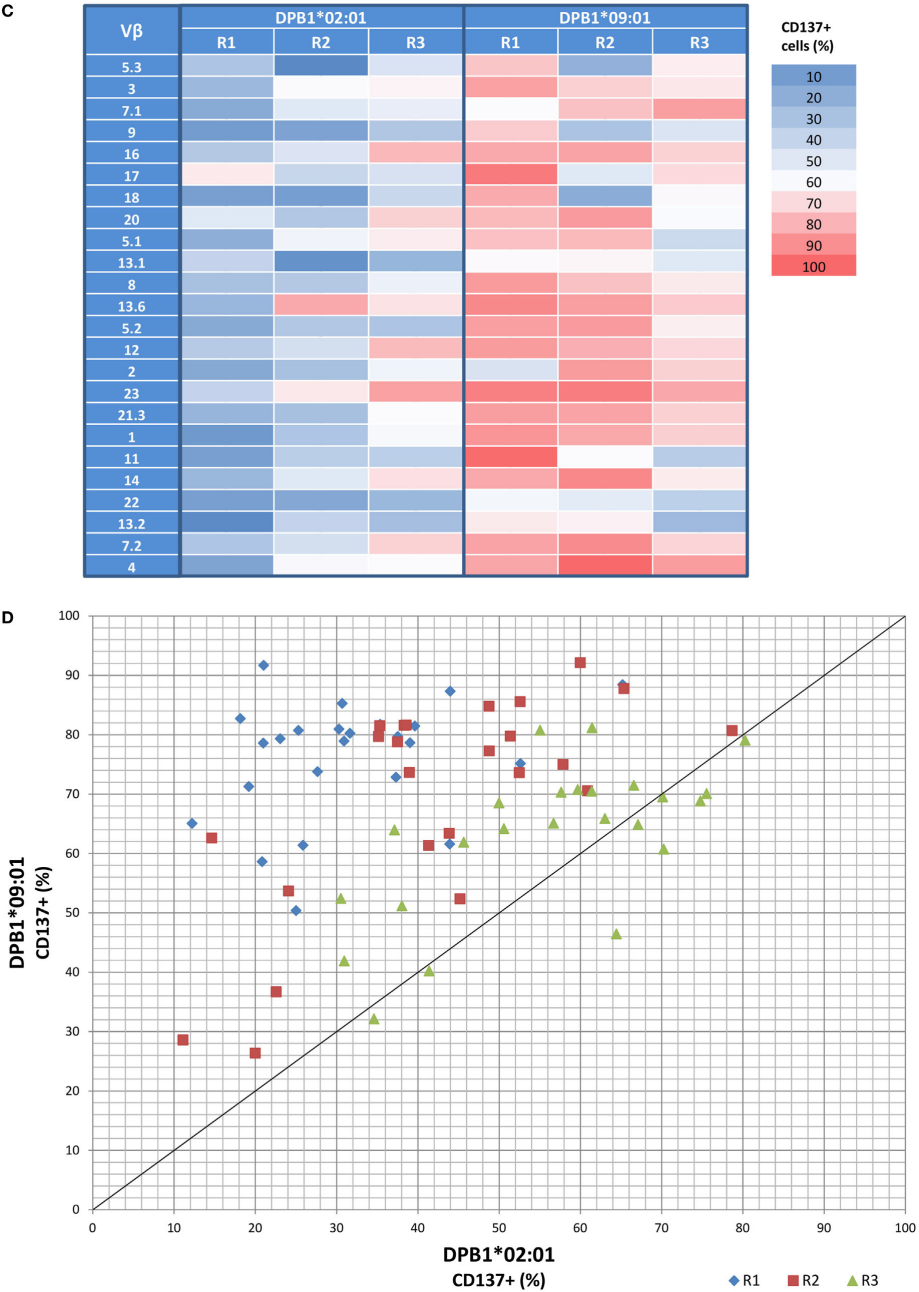
Alloresponses against both HLA-DPB1*02:01 and DPB1*09:01 originate from a varied repertoire at the Vβ level, but with stronger responsiveness against the latter. TCR-Vβ analysis of 24 Vβ specificities was performed by flow cytometry in CD4+ T cells from three self-DPB1*04:01,*04:02 responders (R) after 2 weeks of culture with HeLa cells expressing DPB1*02:01 or DPB1*09:01, followed by overnight reincubation with HeLa cells expressing the original alloantigen. **(A)** Percentage of cells pre-culture and in the CD137+ cultured fractions after restimulation expressing each of the targeted Vβ families against each alloantigen. **(B)** Enrichment in the CD137+ cultured fraction vs. pre-culture levels (*y* axis) of each of the Vβ specificities was quantified and plotted against CD137 positivity for all cells expressing that Vβ family after restimulation (*x* axis). The size of each circle represents the share (% cells positive) of each Vβ specificity in the CD137+ T cell receptor repertoire. Vβ family dynamics against DPB1*02:01 (blue circles) and DPB1*09:01 (red circles) are shown for each responder. **(C)** Heat map showing CD137 positivity levels among all cells expressing each Vβ specificity for each of the responders against the two DPB1 alleles. Shown in red are strongly responding Vβ specificities (>60% CD137+ cells after restimulation among all CD4+ cells expressing that specificity). **(D)** Plot showing the response (CD137 levels) of each of the targeted Vβ specificities against DPB1*02:01 (*x* axis) and DPB1*09:01 (*y* axis) for each of the three responders. Each dot represents one targeted Vβ specificity for each of the responders. The majority of the Vβ specificities respond with higher CD137 levels against DPB1*09:01 than to DPB1*02:01.

We first analyzed the TCR diversity among these cells at the level of Vβ families by using flow cytometric quantification (Figure 2). We observed that all Vβ specificities tested could be found among the DPB1*02:01 and DPB1*09:01-reactive CD4+ cells (Figure 2A) and that the frequency of the majority (12–16/24 against DPB1*02:01; 12–20/24 against DPB1*09:01) of targeted Vβ specificities expanded during culture, with average fold expansions of 3.86 against DPB1*02:01 and 3.50 against DPB1*09:01. There was a correlation between CD137 positivity in each family and fold expansion from pre-culture levels, with responses against DPB1*09:01 showing a shift to higher levels of CD137 positivity (Figure 2B). This difference was also reflected in the number of highly reacting Vβ specificities (i.e., Vβ for which >60% of all cells expressed CD137 after restimulation) against each allele: 1–11/24 against DPB1*02:01 vs. 18–22/24 against DPB1*09:01 (Figure 2C). In addition, in 63/72 cases the same Vβ family responded with higher CD137 levels against DPB1*09:01 than to DPB1*02:01 (overall average 26.1% higher) (Figure 2D). The cumulative frequency of the top 10 Vβ specificities was similar for both alleles (63.8% against DPB1*02:01 vs. 61.7% against DPB1*09:01). Overall, these data at the Vβ level suggest that responses against a single or multiple amino acid differences in HLA-DP arise from comparable levels of diversity, albeit with overall lower levels of alloreactivity against the more similar allele.

### Clonality and Richness of TCR Clones Responding against a Single or Multiple Amino Acid Differences between DPB1 Alleles

**Figure 3:**
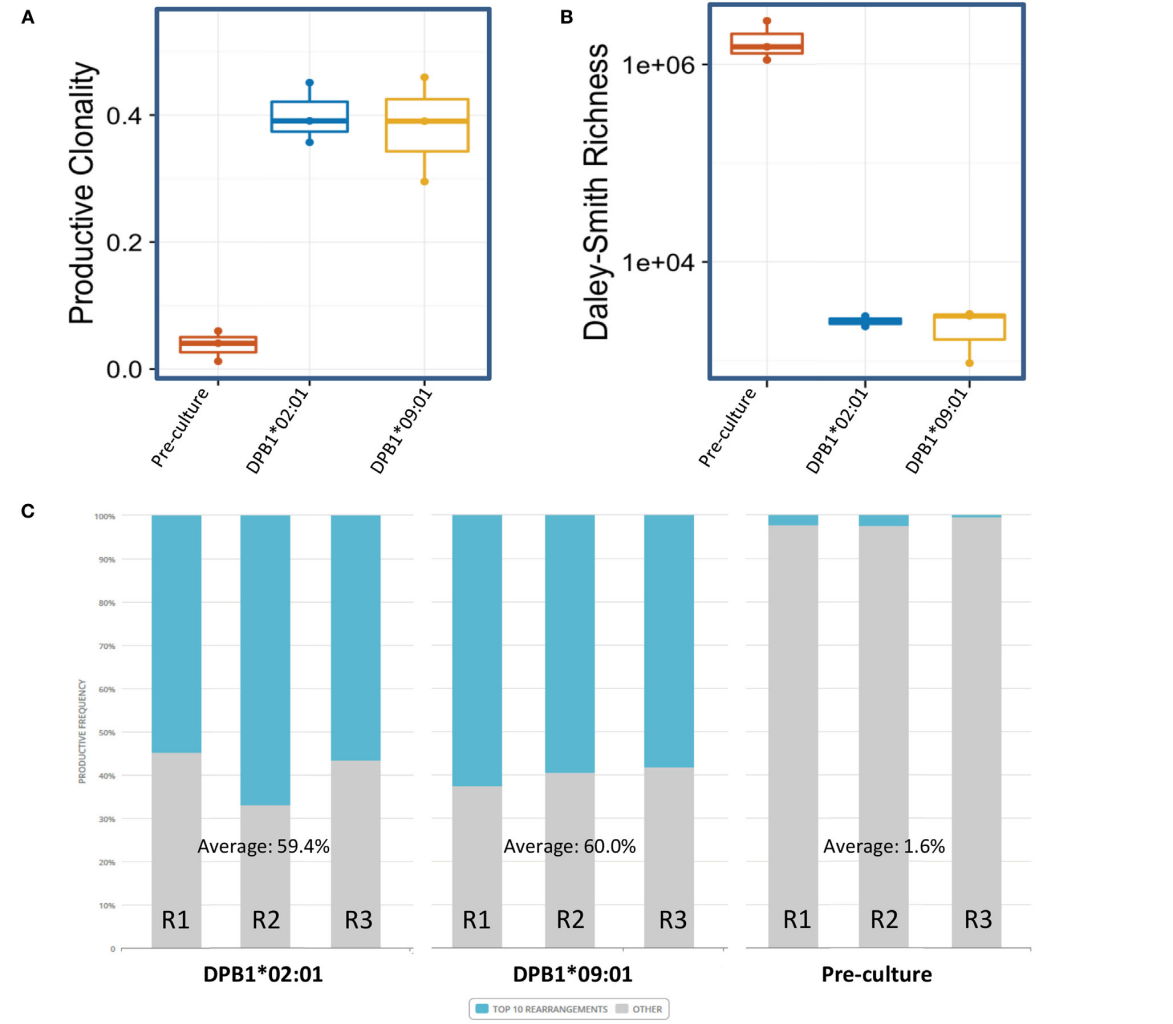

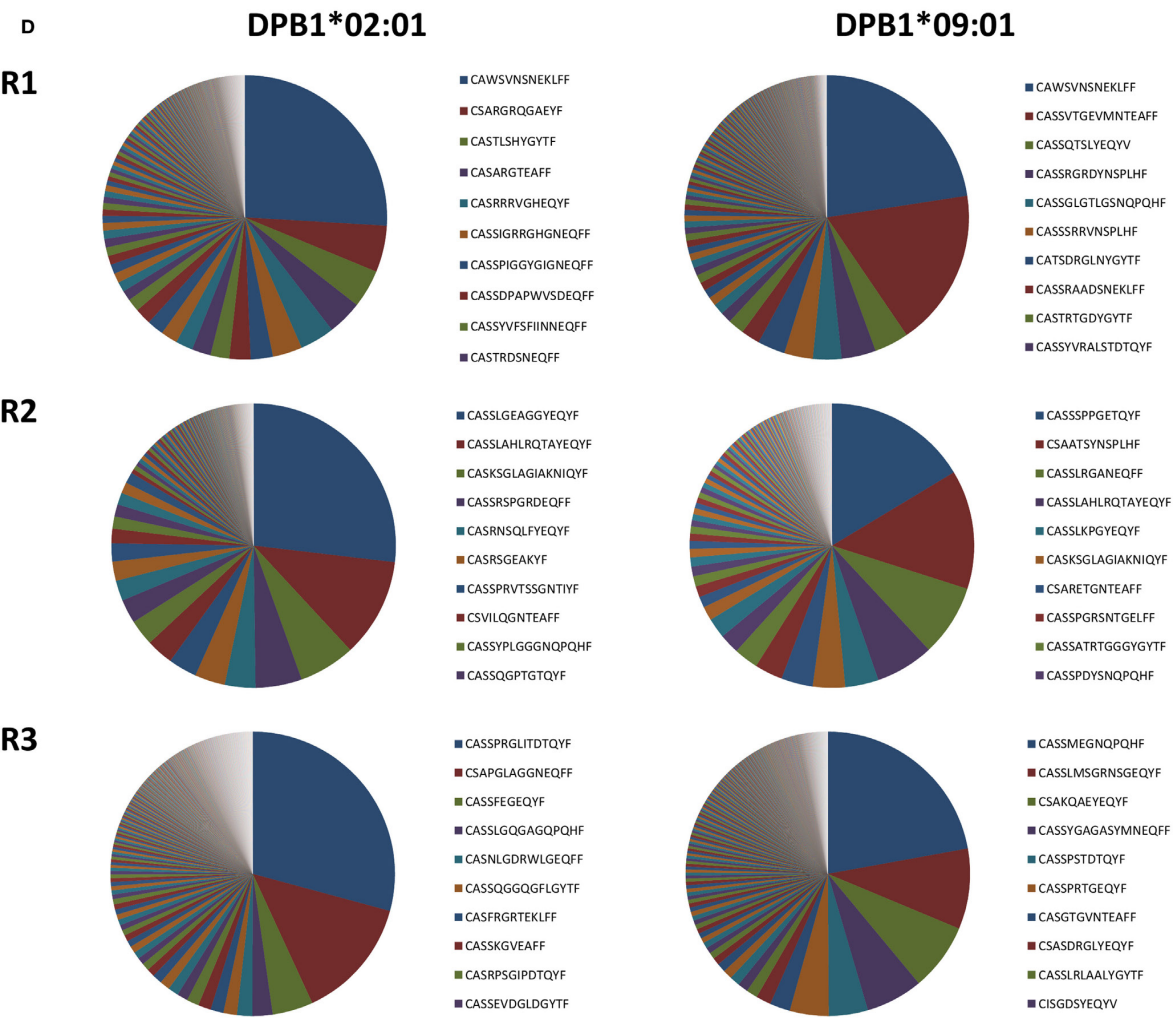
Diversity of CD4+ T cell responses against DPB1*02:01 and DPB1*09:01 at the CDR3 level. Immunosequencing of *TCRB* of sorted CD4+CD137+ cells was performed, and the T cell receptor (TCR) repertoire diversity measures were quantified in CD4+ T cells from three self-DPB1*04:01,*04:02 responders (R) after 2 weeks of culture with HeLa cells expressing DPB1*02:01 or DPB1*09:01, followed by overnight reincubation with HeLa cells expressing the original alloantigen. Median and interquartile ranges for **(A)** productive clonality and **(B)** richness of TCR clones from each responder are plotted, including pre-culture CD4+ cells. **(C)** Cumulative frequency of the top 10 clones (blue) for each set of alloreactive clones and the pre-culture TCR repertoire is shown. Top 10 clones at the DNA (or amino acid in brackets) level accounted for 54.73% (56.04%), 66.83% (68.71%), and 56.56% (58.85%) against DPB1*02:01, and 62.5% (63.21%), 59.5% (63.99%), and 58.14% (60.80%) against DPB1*09:01 for R1, R2, and R3, respectively. Pre-culture top 10 clones at the DNA (or amino acid in brackets) level accounted for 2.14% (2.20%), 2.39% (2.45%), and 0.29% (0.31%) for R1, R2, and R3, respectively. **(D)** Pie plots showing the frequency distribution for clones at the amino acid level responding against DPB1*02:01 (left plots), and DPB1*09:01 (right plots) for each responder. Listed are the top 10 clones for each sample. The total number of clones at the amino acid level was 481, 409, and 406 against DPB1*02:01, and 761, 170, and 700 against DPB1*09:01 for R1, R2, and R3, respectively.

**Figure 4:**
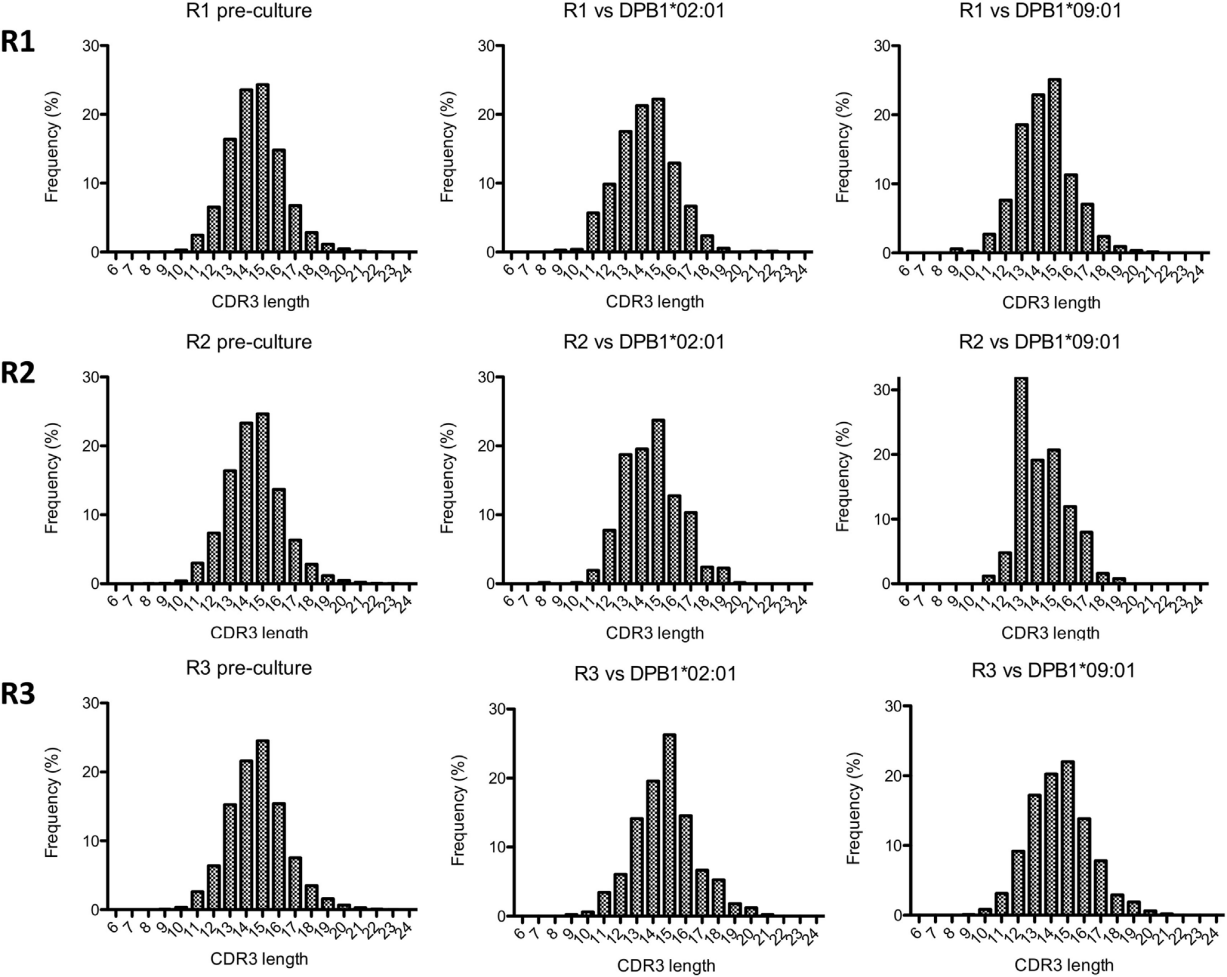
Complementarity-determining region 3 (CDR3) length analysis of alloresponses against DPB1*02:01 and DPB1*09:01. The length of CDR3 regions (number of amino acids) as determined by immunosequencing for each responder’s (R) samples was quantified. Shown are histograms for the total pre-culture CD4+ cells (left panels) and cultured CD4+CD137+ cells alloreactive against DPB1*02:01 (middle panels) and DPB1*09:01 (right panels).

We then analyzed the diversity of alloreactive DPB1-specific CD4+ T cells at the CDR3 level by *TCRB* immunosequencing. Table 2 shows the summary data for the immunosequencing results. As expected, clonality for the cultured CD137+CD4+ cells (mean 0.39) was higher than in pre-culture samples (average 0.04). However, the diversity among T cell clones (as defined by their CDR3 sequence at the amino acid level) responding against DPB1*02:01 was comparable to those responding against DPB1*09:01, and mean productive clonality of TCR clones responding against DPB1*02:01 (0.40, range 0.36–0.45) was similar to that of the clones responding against DPB1*09:01 (0.38, range 0.30–0.46) (Figure 3A). The clone richness was also markedly reduced when compared to the pre-culture samples, but did not differ substantially between clones responding against either allele (Figure 3B). The share for the top 10 reactive clones in the cultured samples ranged from 54.7 to 66.8%, with no substantial difference between cultures responding against DPB1*02:01 (59.4%, range 58–62%) or DPB1*09:01 (mean 60.0%, range 55–67%) (Figure 3C). In accordance with these results, the frequency distribution of T-cell clones did not show any major difference between alloresponses against these two alleles (Figure 3D), with similar number of high-frequency clones (≥1%): 20, 19, 13 against DPB1*02:01; 11, 17, 13 against DPB1*09:01 for R1, R2, and R3, respectively. Finally, analysis of CDR3 length among alloreactive clones revealed no major skewing in the responses against either allele in comparison to pre-culture samples (Figure 4). Overall, these data correlate with the Vβ analyses and suggest that the responding TCR clones elicited *in vitro* against a single or multiple amino acid differences in DPB1 do not differ substantially in terms of their size and diversity.

**Table 2:** Immunosequencing data for pre-culture and cultured samples.

Sample	Responder (R)	Number of productive templates (total T cells)	Number of rearrangements (unique T cells)	Productive clonality	Maximum clonal frequency (%)
Pre-culture CD4+	R1	180,066	98,044	0.0599	0.38
	R2	167,264	116,092	0.0406	0.53
	R3	168,885	146,063	0.0122	0.04

DPB1*02:01-specific CD137+CD4+ cells	R1	7,543	720	0.3909	25.76
	R2	7,874	619	0.4512	26.35
	R3	2,530	495	0.3572	27.94

DPB1*09:01-specific CD137+CD4+ cells	R1	26,837	1,175	0.4596	22.51
	R2	1,447	251	0.2954	14.93
	R3	9,927	959	0.3905	21.80

### Overlap of TCR Clones Responding against DPB1 Alleles

**Figure 5:**
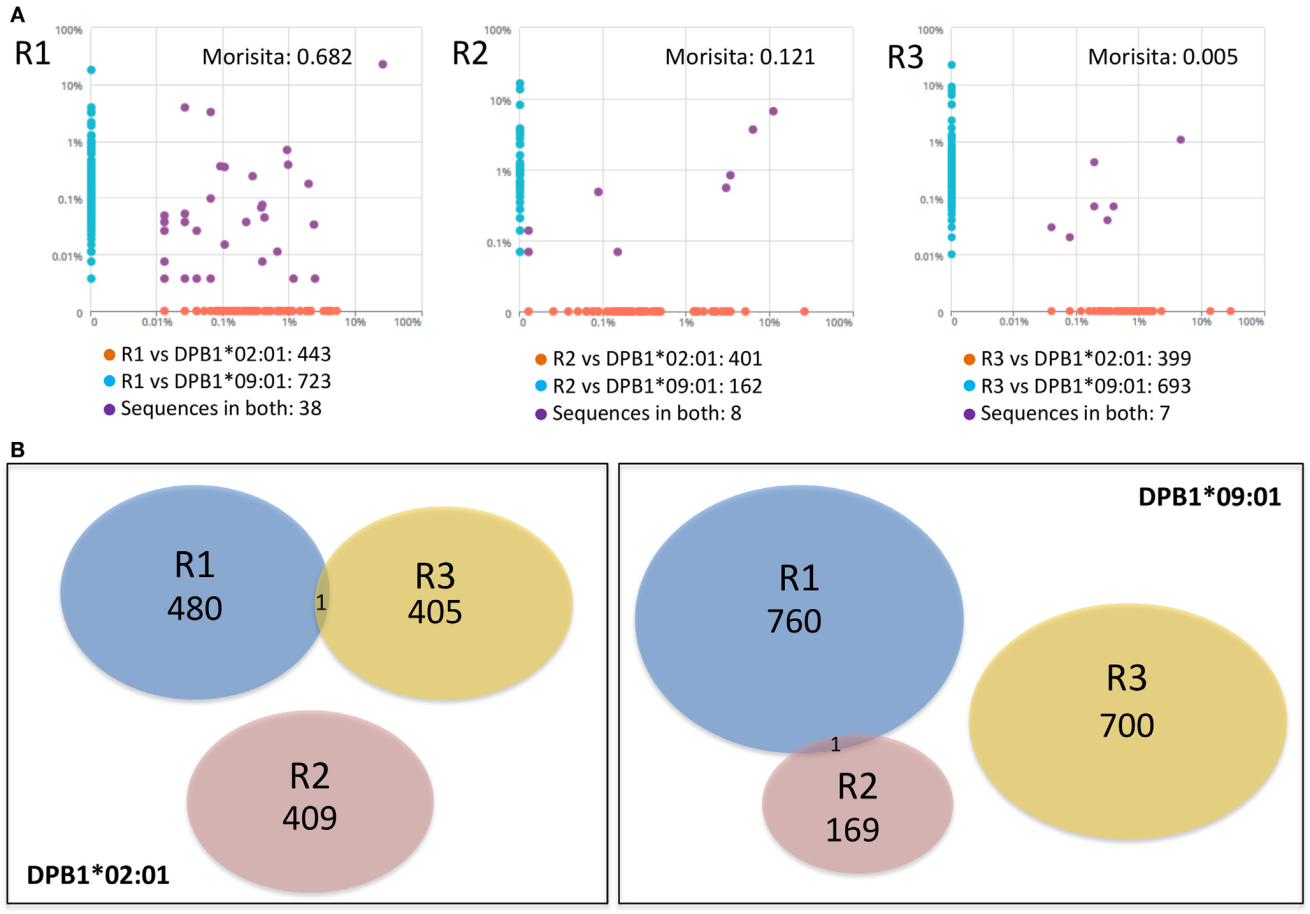
Responses against DPB1*02:01 and DPB1*09:01 alleles are highly divergent and individualized. The sharing of *TCRB*-CDR3 amino acid sequences among CD4+CD137+ cells responding against DPB1 alleles was compared. **(A)** Pairwise scatter plots showing the frequency and sharing of clones against each allele in each of the three responders (R). Within each responding individual, there was a low sharing of clones (purple dots) between their responses against DPB1*09:01 (*y* axis) and DPB1*02:01 (*x* axis). Morisita’s indexes for each pairwise T cell receptor clone set comparison are shown. **(B)** Venn diagrams show the sharing of clones across individuals against DPB1*02:01 (left panel) and DPB1*09:01 (right panel). The number of unique sequences in each clone set is depicted. Only one DPB1-alloreactive clone was shared across individuals for each allele.

We then asked whether there was overlap between individual clone sets responding against either allele and between clones responding against the same allele across individuals. Overall repertoire similarity among cultured samples was low (mean Morisita’s index 0.054). There was little overlap between clone sets responding against DPB1*02:01 and DPB1*09:01 within the same individual [median Morisita’s index: 0.12 (0.005–0.68)] (Figure 5A), and almost no overlap against the same allele across individuals (Figure 5B). At the amino acid level, each individual’s clones sets reactive against DPB1*02:01 and DPB1*09:01 shared only 38/1,204, 8/571, and 7/1,099 sequences (55/1,840, 13/857, and 7/1,447 nucleotide sequences) (Figure 5A). Analysis of the 10 most frequent sequences revealed almost no sharing between cultures (Table 3; Figure 3D). Apart from a single nucleotide sequence shared between two individuals responding against DPB1*02:01 and two clones against DPB1*09:01 with the same amino acid sequence but different nucleotide sequences, no clone was shared by subjects responding against the same allele (Figure 5B). Overall, these data suggest that, despite showing similar repertoire size metrics, responses against these DPB1 alleles are highly divergent, while responses against the same allele are highly individualized.

**Table 3:** Top 10 CDR3 amino acid sequences for CD137+CD4+ cells.

DPB1*02:01-specific CD137+CD4+ cells	Amino acid sequence	Frequency in cultured sample (%)	Frequency in pre-culture sample (%)
R1	CAWSVNSNEKLFF	25.9446	0.0405
	CSARGRQGAEYF	5.2897	ND
	CASTLSHYGYTF	4.3219	ND
	CASARGTEAFF	3.9639	ND
	CASRRRVGHEQYF	3.9374	0.0128
	CASSIGRRGHGNEQFF	3.3674	0.0006
	CASSPIGGYGIGNEQFF	2.5321	0.0006
	CASSDPAPWVSDEQFF	2.3996	0.0017
	CASSYVFSFIINNEQFF	2.1477	0.0033
	CASTRDSNEQFF	2.1344	0.0006

R2	CASSLGEAGGYEQYF	26.8732	0.0018
	CASSLAHLRQTAYEQYF	11.2395	0.5548
	CASKSGLAGIAKNIQYF	6.4135	0.0006
	CASSRSPGRDEQFF	5.2705	ND
	CASRNSQLFYEQYF	3.4798	ND
	CASRSGEAKYF	3.4417	0.0048
	CASSPRVTSSGNTIYF	3.2512	ND
	CSVILQGNTEAFF	3.0734	0.0012
	CASSYPLGGGNQPQHF	2.9337	0.0006
	CASSQGPTGTQYF	2.7305	ND

R3	CASSPRGLITDTQYF	29.2095	0.0006
	CSAPGLAGGNEQFF	13.9130	0.0006
	CASSFEGEQYF	4.6245	0.0012
	CASSLGQGAGQPQHF	2.2925	0.0012
	CASNLGDRWLGEQFF	1.7391	ND
	CASSQGGQGFLGYTF	1.5415	ND
	CASFRGRTEKLFF	1.4625	ND
	CASSKGVEAFF	1.4625	0.0006
	CASRPSGIPDTQYF	1.3834	ND
	CASSEVDGLDGYTF	1.2253	0.0006

DPB1*09:01-specific CD137+CD4+ cells	**Amino acid sequence**	**Frequency in cultured sample (%)**	**Frequency in pre-culture sample (%)**

R1	CAWSVNSNEKLFF	22.5659	0.0405
	CASSVTGEVMNTEAFF	17.93	0.0006
	CASSQTSLYEQYV	3.9200	ND
	CASSRGRDYNSPLHF	3.8976	0.0061
	CASSGLGTLGSNQPQHF	3.2530	0.0006
	CASSSRRVNSPLHF	3.2455	0.0072
	CATSDRGLNYGYTF	3.1337	0.0028
	CASSRAADSNEKLFF	2.1426	0.0061
	CASTRTGDYGYTF	1.8556	ND
	CASSYVRALSTDTQYF	1.2632	ND

R2	CASSSPPGETQYF	16.3787	0.0036
	CSAATSYNSPLHF	13.5453	ND
	CASSLRGANEQFF	8.1548	0.0006
	CASSLAHLRQTAYEQYF	6.6344	0.5548
	CASSLKPGYEQYF	3.8010	ND
	CASKSGLAGIAKNIQYF	3.6628	0.0006
	CSARETGNTEAFF	3.5936	ND
	CASSPGRSNTGELFF	3.1790	ND
	CASSATRTGGGYGYTF	2.7643	0.0042
	CASSPDYSNQPQHF	2.2806	0.0018

R3	CASSMEGNQPQHF	22.1416	ND
	CASSLMSGRNSGEQYF	9.1468	0.0024
	CSAKQAEYEQYF	7.6760	ND
	CASSYGAGASYMNEQFF	6.4874	0.0006
	CASSPSTDTQYF	4.4525	0.0065
	CASSPRTGEQYF	4.4223	ND
	CASGTGVNTEAFF	2.3169	ND
	CSASDRGLYEQYF	1.7024	0.0006
	CASSLRLAALYGYTF	1.2894	ND
	CISGDSYEQYV	1.1685	0.0006

*CDR3, complementarity-determining region 3; ND, not detected*.

### Clonal Enrichment and Abundance of TCR Clones Reacting against a Single or Multiple Amino Acid Differences between DPB1 Alleles

**Figure 6:**
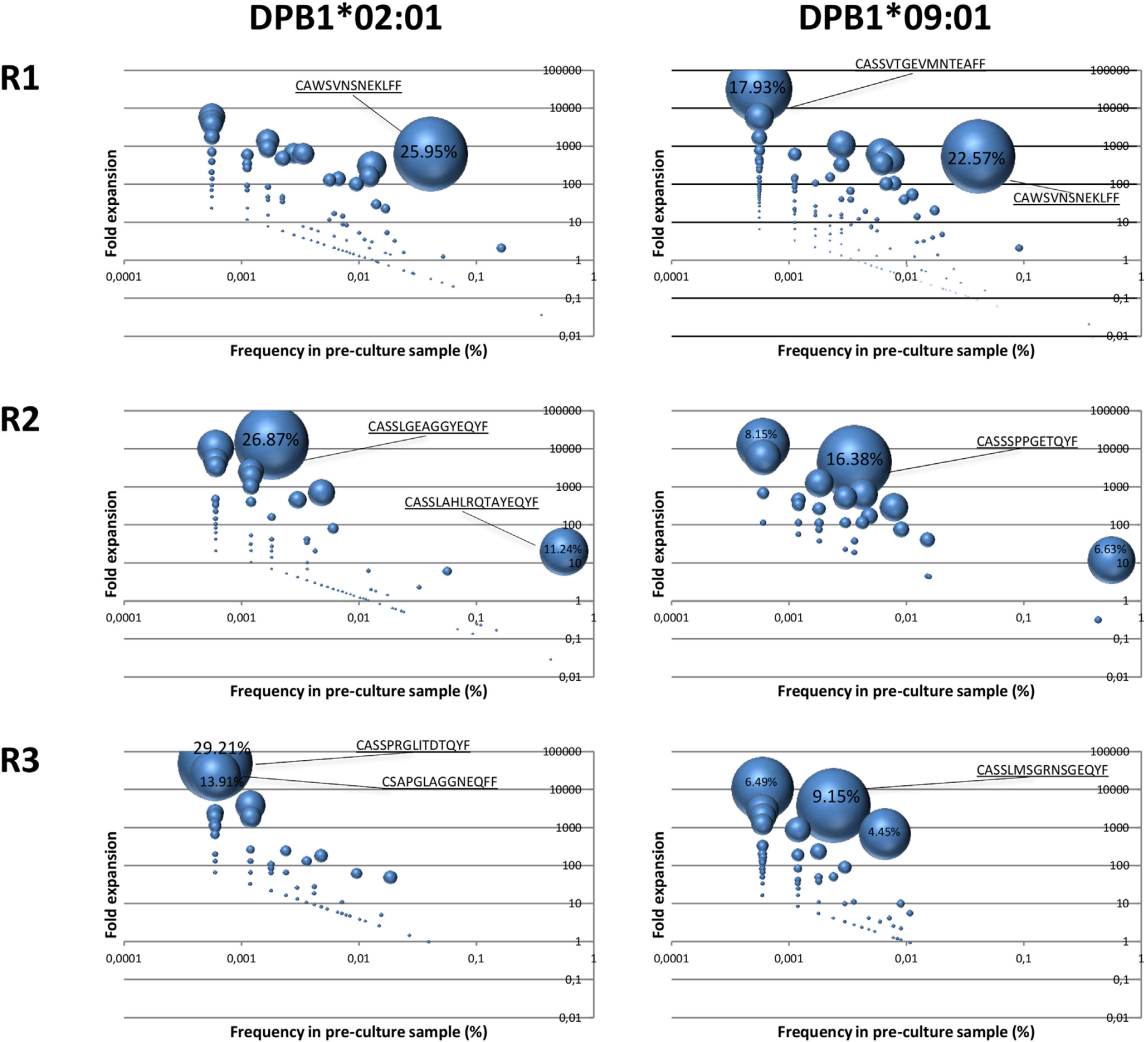
Expansion of DPB1-specific alloreactive clones detected in pre-culture samples. Fold expansion among alloreactive T cell clones present in the cultured samples and that could be detected in the pre-culture samples for each responder (R) was quantified and plotted (*y* axis) against their frequency in the pre-culture sample (*x* axis). The size of the circle represents the share of each clone in the total cultured set. Highest values (%) are indicated for each sample. Left panels, clones responding against DPB1*02:01; right panels, clones responding against DPB1*09:01. As shown in Figure 7A (purple dots), 177, 146, and 92 clones against DPB1*02:01, and 249, 43, and 112 clones against DPB1*09:01 were detected in both the cultured and the pre-culture samples for R1, R2, and R3, respectively.

In order to assess clone expansion against a single or multiple amino acid differences in DPB1, we examined their kinetics in terms of enrichment (25) and pre-culture abundance. Significantly enriched clones in the cultured samples with respect to the pre-culture repertoires accounted for a large part of the clones responding against DPB1*02:01 (mean cumulative frequency 82.4%, range 71.82–89.26%). This was similar against DPB1*09:01 (mean 84.2%, range 74.5–93.3%). A total of 69, 52, and 33 clones against DPB1*02:01 and 172, 26, and 105 clones against DPB1*09:01 showed statistically significant enrichment in cultured samples (*p* < 0.01). For significantly enriched clones detected in the pre-culture samples, average fold expansions against DPB1*02:01 were 866× (range 15–6,016×), 2,245× (6–14,693×), and 5,350× (49–47,194×), for R1, R2, and R3, respectively, while among clones responding against DPB1*09:01 they were 873× (2–32,260×), 2,012× (11–12,022×), and 1,396× (6–10,786×) for R1, R2, and R3, respectively. As shown in Figure 6, among clones detected in both the cultured and the pre-culture samples, apart from a few cases where higher frequency clones expanded strongly, those with lower pre-culture frequencies constitute a large part of the cultured clones, with no major difference between DPB1 alleles.

**Figure 7:**
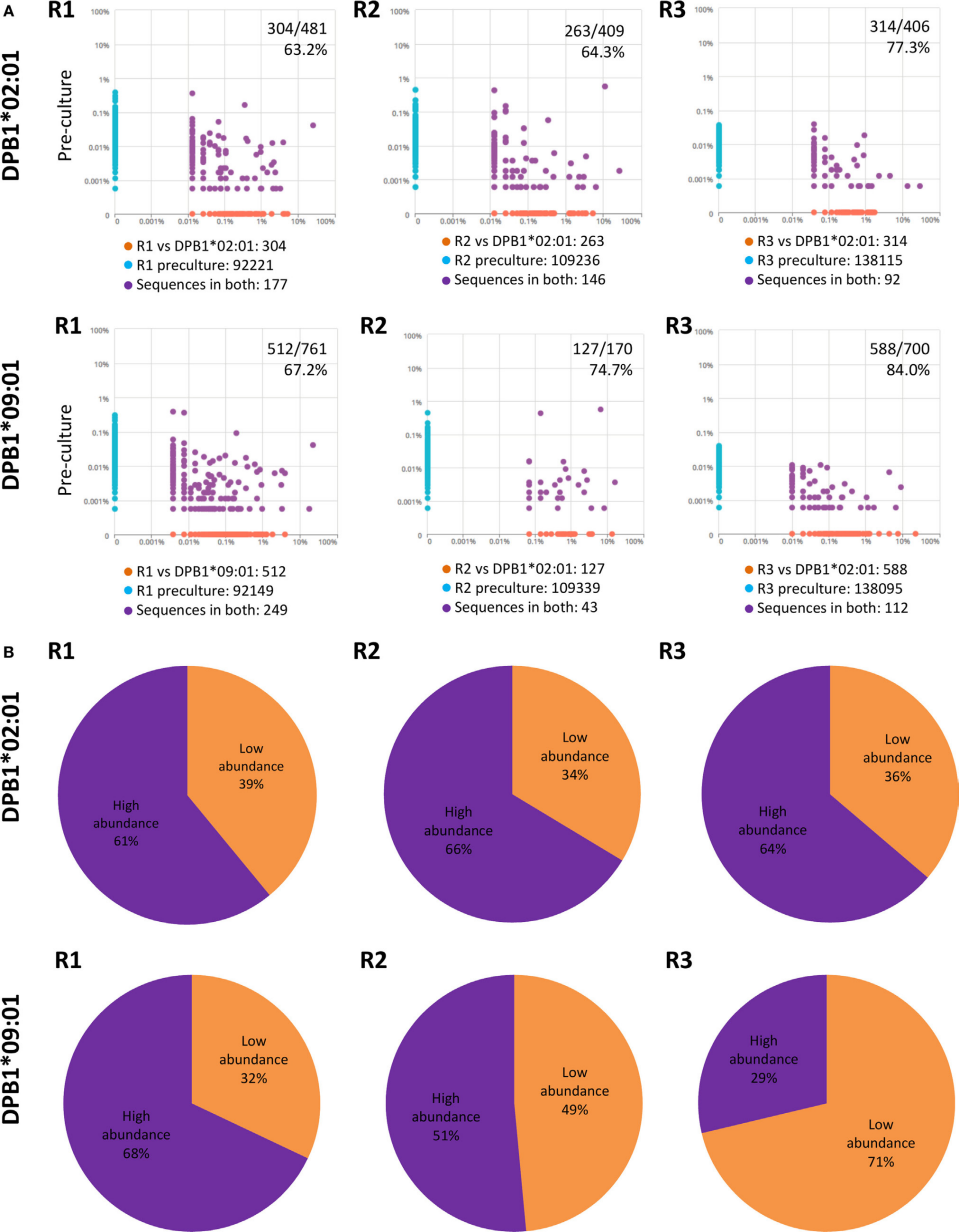
Low abundance pre-culture T cell clones represent a substantial part of the alloreactive clones elicited against HLA-DPB1 molecules with higher share against DPB1*09:01. **(A)** Clone (CDR3 amino acid) pairwise scatter plots between pre-culture repertoires and CD4+CD137+ cells responding against DPB1*02:01 (top panels) and DPB1*09:01 (bottom panels) are shown for each responder (R). Low-abundance DPB1-alloreactive clones (orange dots in the pairwise scatter plots) were defined as those present in the cultured sample (*x* axis) and not detected in the respective pre-culture repertoire (*y* axis). The number and percentage of low abundance clones relative to the total number of clones detected in the cultured sample are shown. **(B)** The share (cumulative frequency) of low abundance clones was quantified for clones responding against DPB1*02:01 (top panels) or DPB1*09:01 (bottom panels) in each subject. The average cumulative frequency for low-abundance clones against DPB1*02:01 and DPB1*09:01 was 36.3 and 50.6%, respectively.

Due to the fact that low-abundance (i.e., rare) alloreactive T cell clones seem to constitute a major component of alloresponses (29), we evaluated their presence and cumulative frequencies among clones responding against DPB1*02:01 and compared them to responses against DPB1*09:01. We defined low-abundance pre-culture clones as those present in the cultured samples but not detectable in the respective pre-culture samples (Figure 7A). We found that low-abundance clones represented the majority of the clones identified against DPB1*02:01 (average 68.3%) and DPB1*09:01 (average 75.3%). However, the mean cumulative frequency of low-abundance clones expanded against DPB1*02:01 was lower in comparison to those expanded against DPB1*09:01 (average 36.3%, range 33.6–39.0% and average 50.6%, range 32.0–71.3%, respectively) (Figure 7B). Overall, these data show that DPB1-specific alloreactive clones arise preferentially from low-frequency clones, with undetected low-abundance pre-culture T cell clones representing a substantial part of the alloreactive repertoires against HLA-DP molecules, in particular against dissimilar DPB1.

## Discussion

In this study, we have characterized the self-selected (i.e., self-DPB1*04:01,*04:02) alloreactive CD4+ T cell clone sets generated against structurally similar (DPB1*02:01) or dissimilar (DPB1*09:01) HLA-DP molecules in healthy individuals using Vβ immunophenotyping and, for the first time, cutting-edge high-resolution immunosequencing of the TCR. Our analyses show that despite quantitative differences in the strength of the *in vitro* alloresponse, with higher levels of reactivity against the more dissimilar allele, a single amino acid difference in the peptide-binding groove encoded by allogeneic DPB1*02:01 compared to self-DPB1*04:02 is able to generate an array of alloreactive TCR clones of similar broadness and diversity to those elicited by 10 amino acid changes in the peptide-binding groove encoded by allogeneic DPB1*09:01, with no major differences in clonality and other diversity measures when comparing both alloantigens. In addition, we show a very low overlap between the responses against both alleles at the individual subject level, and essentially no overlap among TCRs responding against the same allele across individuals. Finally, by making use of the unique power of immunosequencing we show that clones with low pre-culture frequencies appear with high frequency among the expanded alloreactive clones and confirm previous observations regarding the relevant role of low-abundance clones in alloreactivity (30). We suggest that this compartment might play a more important role in alloreactivity to more dissimilar DPB1 alleles based on their greater share of the alloreactive clone array elicited by DPB1*09:01 compared with DPB1*02:01.

By using specific DPB1 mismatches (i.e., DPB1*04:01,*04:02 vs. DPB1*02:01 or DPB1*09:01) involving a single or multiple amino acid changes in the HLA-DP peptide-binding groove with respect to one of the self-selecting alleles (i.e., DPB1*04:02), we sought to dissect the effect of the extent of variation in this region of the HLA-DP molecule exerted indirectly *via* thymic selection on the diversity of clones expanded from the alloreactive TCR repertoire. DPB1*09:01 and DPB1*04:01/*04:02 differ at 13 and 10 positions in exon 2, respectively. It is expected that the peptide repertoires of these molecules differ substantially, providing a plausible explanation for strong alloreactivity and diverse TCR responses observed among self-DPB1*04:01,*04:02 individuals. On the other hand, the DPB1*02:01 exon 2 differs from DPB1*04:02 only at position 69 (E69K). This makes it likely that the peptide repertoires of these two molecules overlap to a certain extent. Indeed, analysis of the peptide binding motifs for DPB1*02:01 (31) and DPB1*04:02 (unpublished data, manuscript in preparation) show that peptide position P4, which interacts with the pocket formed in part by the side chain of position 69 on the alpha-helix of the DP beta polypeptide, is the only position that seems to differ significantly between these two alleles, with DPB1*02:01 having affinity for lysine and DPB1*04:02 for glutamic acid at P4. Importantly, position 69 on the DPB1*02:01 molecule has been shown to impact the recognition of this molecule by monoclonal antibodies (32), bone marrow recipient–donor mixed-lymphocyte reactions (10), and the lysis of DPB1*02:01-expressing cells by alloreactive T cell clones (33, 34). Moreover, mutation of position 69 in DPB1*02:01 was shown to impact the class II-associated invariant chain-derived peptide binding affinities of pockets 4 and 6 (35). Interestingly, this position of the DPB1 molecule and the specific amino acid E69K change have been previously identified as having a strong impact on the generation of alloreactivity against DPB1*09:01 (13).

Of note, some studies have reported that class I HLA molecules with numerous sequence differences do not elicit an allogeneic cytotoxic lymphocyte response (36) and that such highly diverged mismatches might be acceptable in HSCT (37). We have not observed this phenomenon in our CD4+ assays, neither with DPB1*09:01 nor with other alleles having several amino acid differences when compared to the autologous alleles (unpublished data). Intuitively, more amino acid differences in the peptide-binding grove would generate more divergent peptide repertoires resulting in a lower indirect effect of thymic selection and higher alloreactivity, something that lies at the basis of the TCE model.

In the context of allogeneic HSCT, compatibility between donor and recipient for polymorphic HLA plays a central role in the balance between the detrimental graft-vs.-host and therapeutic graft-vs.-leukemia effects (38, 39), both mediated mainly by alloreactive T cell responses. Because of this, the search of HLA permissive mismatches, which contribute to cure the patient’s malignancy while reducing the toxicity of the transplant, is a major goal in HSCT (40). Permissiveness to DPB1 mismatches is now a well-established phenomenon in HSCT, both clinically (14, 15) and *in vitro* (9, 12, 41), with permissive mismatches (i.e., those involving two alleles of the same T cell epitope, TCE, group, and that share structural similarities) conferring significantly lower risks of relapse without significant increases in non-relapse mortality when compared to DPB1 allele matches after HSCT for hematologic malignancies (14, 42, 43).

Clinically, a mismatch involving a donor who is self-DPB1*04:01,*04:02 and a patient bearing DPB1*02:01 would currently be considered permissive, whereas one involving a patient with a self-DPB1*09:01 is considered non-permissive. Our data show that, contrary to the strength of the alloreactive response, the diversity of the alloreactive clones elicited by such a permissive mismatch does not seem to be impacted by the number of amino acid differences in the peptide-binding groove. This feature is desirable for the therapeutic effect of HSCT: moderate alloreactivity levels combined with a sufficiently broad repertoire maximizing the capacity of donor-derived T cells of effectively recognizing malignant patient cells while minimizing GvHD.

Since permissiveness was not predicted by significant differences in terms of the size of the set of responding TCR clones, it is possible that a qualitative characteristic of the HLA–TCR interaction influencing events downstream of TCR recognition (44, 45) and shaped by thymic selection might be responsible for this phenomenon (16, 46). Thymic education in a self-DPB1*04:01,*04:02 individual could have an indirect effect on the allogeneic repertoire capable of reacting to a structurally related molecule such as DPB1*02:01, by reducing the affinity of the binding to the allogeneic molecule. This would not result in more restricted reacting TCR repertoires but in lower activation and proliferation of the T cells. We cannot, however, rule out that TCR repertoire breadth plays a role in permissiveness to other specific allelic combinations. Moreover, DPB1*02:01 might still represent a separate TCE group (47) and hence behave differently with respect to other alleles sharing similarity to DPB1*04:02. More research into this question is warranted.

Interestingly, low-abundance T cell clones, which have been shown to constitute a significant amount of the alloreactive response *in vitro* (29) and *in vivo* (30), seem to represent a higher proportion of the TCR clone sets against DPB1*09:01 than against DPB1*02:01. A possible explanation for this observation could be that a more functionally distant allele such as DPB1*09:01 might elicit stronger TCR signaling that could help to beat the threshold to extract these specificities from the deep pre-culture repertoire thanks to better proliferative signals.

Recent reports have shown a potential effect of differential 3′UTR-controlled expression levels on permissiveness of DPB1 mismatches in the context of HSCT (48, 49). This model would not play a role in the results presented in this study since the HeLa cell system utilized does not include the 3′UTR region of this gene (18). However, we cannot dismiss a potential effect of this model on the alloreactive TCR repertoires against HLA-DP molecules in a physiological setting.

Our study is limited by the number of subjects included, the use of the HeLa system (i.e., of a non-physiological antigen presenting cell to stimulate our alloreactive T cells), and the use of total CD4+ responder T cells. Although we have been unable to identify any “public” TCR clones responding against DPB1*02:01 and/or DPB1*09:01, a larger study with a significantly larger number of subjects would be required to rule out the existence of such clones in the population. Similarly, the analysis of a potential effect of responder age and the frequency of memory CD4+ T cells in their repertoire on the clonality of HLA-DP alloreactive cells would require a larger number of samples. The use of HeLa cells transduced to express specific DPB1 alleles, although extremely useful experimentally, could skew our results due to the underlying peptidome of this non-hematopoietic tumor cell line. Moreover, we have not addressed the question whether the naïve and memory CD4+ repertoires could behave differently in alloresponses, something that has been previously suggested (50, 51). In addition, despite the high level of over 90% purity of our CD137+ samples, a confounding effect of contaminating CD137− cells especially on rare CDR3 sequences cannot be ruled out. Finally, CD4+ T cells reactive against HLA class I molecules have been described (52, 53). However, their impact on our results, if any, must be minimal, since very low levels of activation were observed when the responder cells were restimulated with the HeLa expressing the autologous DPB1 allele, which would express the same class I molecules as those transduced with the allogeneic molecules.

In conclusion, our study, the first one to comparatively address TCR diversity in responses to similar or dissimilar allogeneic HLA-DP molecules by NGS immunosequencing, shows proof-of-principle evidence for the novel concept that limited strength alloreactivity can coexist with broad TCR diversity, providing a potential platform for clinically favorable DPB1 mismatches in allogeneic HSCT. The potential role for *in vitro* pre-transplant low-abundance clones, revealed solely through the power of NGS immunosequencing, as personalized clinical biomarkers in HSCT needs to be further clarified by *in vivo* tracking of expanded clones in patients with alloreactivity against DPB1 mismatches.

## Ethics Statement

This study was carried out in accordance with the recommendations of University Hospital Essen with written informed consent from all subjects in accordance with the Declaration of Helsinki. The protocol was approved by the local Ethics Committee of University Hospital Essen.

## Author Contributions

EA-B, PC, and KF designed the study; EA-B, PC, MM, and TM performed experiments; MA contributed advice on TCR immunosequencing experiments; PB and JF produced the transduced HeLa cells; EA-B, JR, MV, and EY performed statistical analysis; PH contributed PBMC from healthy blood donors and their HLA typing; and EA-B and KF wrote the manuscript.

## Conflict of Interest Statement

JR, MV, and EY have employment and equity ownership with Adaptive Biotechnologies. All other authors declare that the research was conducted in the absence of any commercial or financial relationships that could be construed as a potential conflict of interest.
